# Supplementary material for: Comparative Transcriptome Analysis Reveals Mechanisms of Differential Salinity Tolerance Between Suaeda glauca and Suaeda salsa
Source: Genes (Basel). 2024 Dec 19;15(12):1628. doi: 10.3390/genes15121628 (PMC11675990; doi:10.3390/genes15121628)
Supplement: Supplementary file 1 [file genes-15-01628-s001.zip › genes-3342618-supplementary.pdf]

Table S1. Statistical analysis of transcriptome sequencing data

BMK-ID	Read Number	Base Number	GC Content	%≥Q30
Sg-CK-L1	22,019,833	6,583,852,458	43.31%	94.03%
Sg-CK-L2	21,196,639	6,337,391,962	43.47%	93.98%
Sg-CK-L3	21,588,127	6,450,336,730	43.52%	94.19%
Sg-CK-R1	21,658,731	6,483,189,432	43.35%	94.25%
Sg-CK-R2	22,434,808	6,712,043,070	43.14%	93.61%
Sg-CK-R3	22,917,251	6,854,023,070	43.19%	93.89%
Sg-T-L1	23,546,532	7,036,002,756	43.06%	94.21%
Sg-T-L2	24,123,349	7,210,720,376	43.05%	94.01%
Sg-T-L3	23,442,661	7,006,546,774	43.45%	94.78%
Sg-T-R1	21,400,984	6,394,340,248	43.25%	94.46%
Sg-T-R2	21,067,045	6,295,988,466	43.26%	94.11%
Sg-T-R3	25,204,526	7,541,805,792	43.05%	94.22%
Ss-CK-L1	22,571,499	6,750,268,496	43.02%	94.11%
Ss-CK-L2	20,662,986	6,181,572,622	43.03%	94.28%
Ss-CK-L3	20,113,146	6,010,961,364	43.02%	94.30%
Ss-CK-R1	21,053,700	6,298,835,750	42.93%	94.41%
Ss-CK-R2	20,169,966	6,029,433,624	42.89%	93.90%
Ss-CK-R3	21,734,075	6,494,715,532	42.91%	94.04%
Ss-T-L1	21,003,449	6,278,158,438	42.91%	94.42%
Ss-T-L2	21,022,696	6,291,175,914	43.07%	94.25%
Ss-T-L3	19,604,138	5,865,190,048	43.06%	94.12%
Ss-T-R1	20,128,506	6,018,026,288	42.84%	94.38%
Ss-T-R2	21,553,035	6,442,421,934	42.66%	94.45%

Table S2. Statistical analysis of the number of differentially expressed genes

DEG Set	DEG number	up-regulated	down-regulated
Sg-CK-L vs Sg-T-L	2,907	1,185	1,722
Sg-CK-R vs_Sg-T-R	9,114	4,758	4,356
Ss-CK-L vs_Ss-T-L	3,419	1888	1,531
Ss-CK-R vs_Ss-T-R	2808	1,317	1,491
Sg-CK-L vs_Ss-CK-L	25,050	12,764	12,286
Sg-CK-R vs_Ss-CK-R	25,449	12,914	12,535
Sg-T-L vs_Ss-T-L	25,539	13,312	12,227
Sg-T-R vs Ss-T-R	26,403	13,954	12,449

Table S3. Statistics of gene expression levels (FPKM) related to ABA biosynthesis and signal transduction, and JA biosynthesis pathways

	Gene ID	Sg-CK-R	Sg-T-R	Ss-CK-R	Ss-T-R
ABA biosynthesis	*ABA4*	1.33	1.203333333	11.42666667	14.05666667
	NCED1	0.29	0.103333333	62.33333333	38.05666667
	ABA2	0.083333333	0.196666667	20.13333333	14.80333333
	AAO3	0.25	0.463333333	11.36	18.86666667
ABA signal transduction	*PYR/PYL*	4.153333333	3.48	38.78666667	16.51
	PP2C59	0.163333333	0.283333333	0.786666667	1.32
	PP2C38	1.626666667	2.263333333	5.673333333	8.243333333
	PP2C27	2.226666667	2.89	22.22666667	25.78666667
	SnRK2	4.89	3.69	78.70333333	89.35666667
	ABF2	2.163333333	1.44	43.58	59.78666667
ERF transcription factor	ERF1	0.263333333	0.443333333	4.19	8.47
	ERF10	0	0.053333333	7.886666667	11.04
	ERF38	0.24	0.233333333	10.07666667	19.05666667
WRKY transcription factor	WRKY13	1.18	0.123333333	25.99666667	45.23333333
	WRKY20	5.35	3.39	24.56333333	31.25333333
	WRKY21	3.37	1.65	50.86333333	60.34
bHLH transcription factor	bHLH49	0.02	0.006666667	13.60666667	21.95333333
	bHLH106	2.356666667	0.243333333	19.5	27.85
	bHLH62	15.31	3.626666667	56.04333333	65.78
	bHLH30	1.493333333	0.04	22.46666667	39.54666667
	bHLH130	0.456666667	0.656666667	6.82	11.64
JA biosynthesis	*LOX3*	5.736666667	27.08	477.97	196.0733333
	AOS1	0.34	0.06	468.0533333	130.71
	AOS1.2	0.213333333	0.166666667	14.35666667	21.17333333
	AOC	3.18	2.896666667	90.63666667	32.40666667
	OPR3	9.206666667	4.873333333	92.07666667	73.92333333
	OPR2	0.033333333	0.06	2.98	7.046666667
	JAR1	2.58	9.513333333	46.69	28.8

Table S4. Statistics of expression levels (FPKM) of candidate genes for salt tolerance in *Suaeda genus* and *Suaeda salsa*

	Gene ID	Sg-CK-R	Sg-T-R	Ss-CK-R	Ss-T-R
Root	*GPAT*	4.516666667	9.996666667	23.7	53.77666667
	GDSL Esterases/Lipases	0.283333333	1.596666667	4.7	24.67333333
	Mannitol dehydrogenase	5.916666667	27.35	36.41	90.71
	KCS11	1.266666667	4.546666667	27.56	65.46333333
	TSJT1	143.2533333	289.6133333	323.4433333	684.83
	HSFs	0.513333333	2.243333333	6.81	91.27333333
	ATPase4	1.376666667	11.41	33.42	73.90666667
	Peroxidase 16	0.056666667	0.883333333	3.646666667	20.9
	NLP9	0.1	0.33	4.266666667	10.88
	ATPase HMA3	0.046666667	0.796666667	3.403333333	8.056666667
	S-adenosylmethionine carrier 1	0.656666667	1.753333333	1.89	4.063333333
	Boron transporter 2	0.923333333	2.543333333	5.983333333	14.89
	PEBP	0.483333333	2.763333333	12.93666667	36.23666667
	Cytochrome P450	0.803333333	2.02	5.086666667	12.41666667
	SBT5.6	0.816666667	1.946666667	11.68	36.77
	SKOR	1.846666667	6.113333333	15.11666667	43.38666667
	SWEET14	0.096666667	4.396666667	4.32	81.43333333
Leaf	*Gene_ID*	Sg-CK-L	Sg-T-L	Ss-CK-L	Ss-T-L
	SWEET17	0.71	1.506666667	9.27	18.92666667
	DIR1	0.27	0.54	18.12333333	346.1466667
	Endochitinase EP3	0.026666667	0.17	3.966666667	13.06333333
	CCCH Zinc-Finger Protein 20	0.36	0.72	61.75	138.3133333
	TLPs 1b	0.16	0.42	2.33	10.84333333
	Galactosyltransferase	1.253333333	3.246666667	7.096666667	17.95
	Glycosyl transferases group 1	0.233333333	0.786666667	11.05	24.96

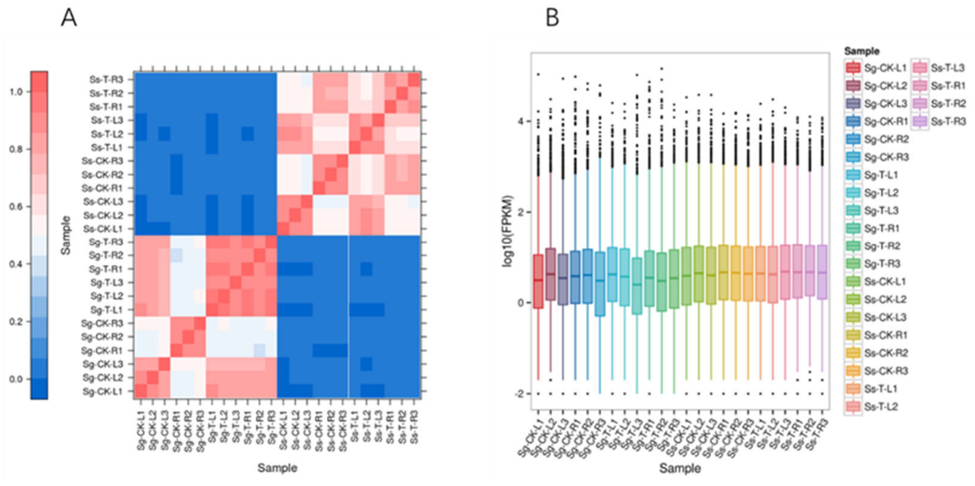

Figure S1. Gene expression level statistics for Unigenes in samples. (A) Correlation analysis of overall gene expression between the roots and leaves of *Suaeda genus* and *Suaeda salsa* under saline-alkali conditions. (B) Box plot representation of gene expression levels (FPKM) across various samples of *Suaeda genus* and *Suaeda salsa*.
